# Metabolomics Analysis for Nitrite Degradation by the Metabolites of *Limosilactobacillus fermentum* RC4

**DOI:** 10.3390/foods11071009

**Published:** 2022-03-30

**Authors:** Chaoran Xia, Qiyuan Tian, Lingyu Kong, Xiaoqian Sun, Jingjing Shi, Xiaoqun Zeng, Daodong Pan

**Affiliations:** 1 State Key Laboratory for Managing Biotic and Chemical Threats to the Quality and Safety of Agro-Products, Ningbo 315211, China; BlackCheshire@163.com (C.X.); b1115132131@163.com (Q.T.); lingyk02645803@163.com (L.K.); dandan5015@126.com (X.S.); sjjing812@163.com (J.S.); daodongpan@163.com (D.P.); 2 Key Laboratory of Animal Protein Food Processing Technology of Zhejiang Province, College of Food and Pharmaceutical Sciences, Ningbo University, Ningbo 315800, China; 3 SinoGrain Linyi DEPOT Ltd. Company, Linyi 276000, China

**Keywords:** lactic acid bacteria, metabolites, nitrite degradation, LCMS

## Abstract

Nitrite (NIT), a commonly used food additive, especially in pickled and cured vegetables and meat products, might cause acute and chronic diseases. Fermentation with lactic acid bacteria (LAB) is an effective method for degrading NIT and improving the flavor of pickled and cured foods. In this study, *Limosilactobacillus fermentum* (*L. fermentum*) RC4 with a high NIT degradation ability was found to degrade NIT in a new manner when compared with reported enzymatic and acid degradation, namely, metabolite degradation during fermentation in MRS broth, which shows a synergistic effect with acid to increase NIT degradation. Liquid chromatography–mass spectrometry analysis identified 134 significantly different metabolites, of which 11 metabolites of *L. fermentum* RC4, namely, γ-aminobutyric acid (GABA), isocitric acid, D-glucose, 3-methylthiopropionic acid (MTP), *N*-formyl-L-methionine, dimethyl sulfone (MSM), D-ribose, mesaconate, *trans*-aconitic acid, L-lysine, and carnosine, showed significant NIT degradation effects compared with the control group (MRS broth). Verification experiments showed that adding the above 11 metabolites to 100 mg/L NIT and incubating for 24 h resulted in NIT degradation rates of 5.07%, 4.41%, 6.08%, 16.93%, 5.28%, 2.41%, 0.93%, 18.93%, 12.25%, 6.42%, and 3.21%, respectively. Among these, three metabolites, namely, mesaconate, MTP, and *trans*-aconitic acid, showed efficient NIT degradation abilities that might be related to the degradation mechanism involving decarboxylation reactions. This is the first systematic study of NIT degradation by LAB, resulting in the identification of a new metabolite degradation pathway and three efficient NIT degradation metabolites.

## 1. Introduction

Nitrite (NIT), one of the most essential food additives due to its excellent color protection, antibacterial [1], and antioxidant properties [2], is mainly used for pickled and cured foods, such as bacon, sausage, ham, and kimchi, to improve the sensory quality, extend the shelf life, and enhance the safety of meat products. NIT can also treat cardiovascular diseases [3]. However, accumulated NIT intake might increase the potential risk of acute and chronic toxicity [4], which has caused consumer concern and reduced pickled and cured food sales. Lactic acid bacteria (LAB), especially *Lactobacillus* sp. cells, are effective in reducing NIT content [5] by producing acid (acid degradation) [6] and nitrite-reducing enzymes (enzymatic degradation). *L. fermentum*, as a probiotic, has great potential in the prevention of human health diseases and in food fermentation and preservation [7]. *L. fermentum* RC4 (CGMCC NO.8212) has been isolated and shows an effective NIT degradation rate of 82% when incubated with NIT (150 mg/L) at 37 °C for 14 h [8]. However, its NIT degradation mechanism has not been studied.

LAB can secrete nitrite reductase [9,10] to degrade NIT at pH > 4.5 [11]. The nitrite reductase system contains NirK, NirS, and NirBD. Among them, NirK and NirS can reduce NIT to nitrogen dioxide, nitrous oxide, or nitrogen under anaerobic conditions through denitrification [12]. In contrast, nitrite reductase (NirBD) degrades NIT to NH_4_^+^ via ammonia [13,14]. Continuous acid production by LAB during fermentation inhibits the activity of nitrite reductases [15,16], with pH becoming the main factor in NIT degradation (pH < 4.5) [8] via a non-enzymatic disproportionation reaction that reduces NIT from NO_2_ to NO [17].

In addition to enzymatic and acid degradation of NIT, LAB metabolites comprise a pathway that might play an important role in NIT degradation, but has yet to be studied systematically. Substances produced by LAB ribosome synthesis mechanisms, such as polypeptides, precursor polypeptides, and proteins, have been shown to reduce the NIT content by inhibiting nitrate reductase activity, reflecting the greater activity at low pH [18]. In vitro, exopolysaccharides (EPS), carbohydrate extracellular compounds metabolized by LAB, were found to significantly scavenge hydroxyl radicals and superoxide radicals [19]. Rather and Zhao found that the NIT clearance rate of acid polysaccharides was higher than that of neutral polysaccharides [20,21]. Furthermore, flavonoids [22,23], phenolic acids [24,25], and hydrogen peroxide [26] have been shown to remove NIT. 

Although the non-enzyme and non-acid metabolites such as exopolysaccharides and polypeptides have been reported to exhibit nitrite-degradation ability, there is no systematic research about NIT degradation for a LAB strain; whether LAB degrades NIT through pathways other than enzymatic and acid degradation remains unclear. Furthermore, the specific substances and their NIT degradation ability are unknown. Therefore, to gain further understanding, this study identified and analyzed the metabolites of *L. fermentum* RC4 from the metabolomics point of view using LC–MS technology and screened the target metabolites for RC4 degradation of NIT to understand the NIT degradation pathway and the detail about metabolites-degradation, which will increase the theoretic knowledge of NIT degradation for LAB.

## 2. Materials and Methods

### 2.1. Bacterial Strains and Growth Conditions

*L. fermentum* RC4 was preserved at the China General Microbiological Culture Collection Center and stored at the State Key Laboratory for Managing Biotic and Chemical Threats to the Quality and Safety of Agro-Products, College of Food and Pharmaceutical Sciences, Ningbo University. *L. fermentum* RC4 was incubated in De Man, Rogosa, and Sharpe (MRS) medium at 37 C for 12 h without shaking to obtain the inoculum prior to use [27].

### 2.2. Preparation of Growth, Acid Production, and NIT Degradation Curves of L. fermentum RC4

NIT powder (Sinopharm Chemical Reagent Co, Shanghai, China) was used to prepare NIT solutions with concentrations of 0–160 mg/L. Weights of 0, 1, 2, 4, 6, 8, 10, 12, and 16 mg NIT powder, respectively, were fixed with pure water in a volumetric flask to 100 mL. NIT standard solutions of 0, 10, 20, 40, 60, 80, 100, 120, and 160 mg/L were obtained.

The nitrite concentrations were determined from a sodium nitrite standard curve [28]. Fresh culture media served as the blank in all experiments. The absorbance at 550 nm was recorded using a spectrophotometer, according to the NIT detection kit (Nanjing Jiancheng Bioengineering Institute, Nanjing, China).

### 2.3. Validation of NIT Degradation by L. fermentum RC4 Metabolites

Sodium lactate buffer solutions of pH 3, 3.5, 4, 4.5, 5, 5.5, and 6 were obtained by mixing lactic acid (Sinopharm Chemical Reagent Co, Shanghai, China) and sodium lactate (Sinopharm Chemical Reagent Co, Shanghai, China) in precise proportions to observe NIT degradation under different pH conditions. NIT (150 mg/L) was prepared and incubated at 37 °C for 48 h.

The RC4 inoculum was cultured in MRS at 37 °C for 30 h. After culture, the MRS broth was immediately centrifuged at 4 °C and 10,000 g for 15 min to remove the bacterium pellet, and then was filtered through a pre-sterilized 0.22-μm membrane filter to harvest the supernatant (pH 4.3). A precise concentration of NIT (ultraviolet irradiation for 30 min as pretreatment) [7] was added to the RC4 supernatant broth and incubated at 37 °C for 24 h. The control group was incubated under similar conditions, without RC4, but at the same pH (4.3). The NIT content was measured every 6 h at 37 °C to determine differences in NIT degradation between the MRS and RC4 metabolites.

*L. fermentum* RC4 supernatant was adjusted with lactic acid–sodium lactate buffer solution and separated into the supernatant fluid (water heating at 100 °C for 15 min, at pH 5.1) and enzyme deactivation (water heating at 100 °C for 15 min, at pH 4.1, 4.6, and 5.6) [10] groups. NIT (13 mg/L; ultraviolet irradiation for 30 min) was added and the NIT content was measured every 5 h for each group. This experiment was used to study NIT degradation by RC4 metabolites.

### 2.4. Liquid Chromatography–Mass Spectrometry (LCMS)-Based Metabolomics of L. fermentum RC4 Fermentation Broth

#### 2.4.1. Metabolite Extraction

Metabolome samples of *L. fermentum* RC4 and MRS broth were prepared, with some modifications, according to a previously described method [29]. To samples of *L. fermentum* RC4 and MRS, we added 60% methanol (*w*/*v*, 15 mL, 20 °C), followed by centrifugation at 8000× *g* for 10 min at 4 °C, transfer to a 1.5-mL centrifuge tube, concentration under vacuum, and freeze-drying. Next, 2-Chlorophenylalanine (4 ppm) and phenylalanine (4 ppm) in methanol were then added to the samples [29], which were then filtered through a 0.22-μm membrane and mixed as quality control (QC) samples, with the remaining sample used for LC-MS detection [30].

#### 2.4.2. Liquid Chromatography and Mass Spectrometry

Liquid chromatography was performed on a Thermo Vanquish UHPLC instrument using an ACQUITY UPLC HSS T3 2.1150-mm 1.8-μm column with an injection temperature of 8 °C. The injection volume was 2 μL, with a flow rate of 0.25 mL/min and column temperature of 40 °C. The mobile phases used in positive and negative ion modes were 0.1% formic acid in water (A1) and 0.1% formic acid in acetonitrile (B1), and 5 mM ammonium formate in water (A3) and acetonitrile (B3), respectively. The gradient elution program was as follows: 0–1 min, 2% B1/B3; 1–9 min, 2–50% B1/B3; 9–12 min, 50–98% B1/B3; 12–13.5 min, 98% B1/B3; 13.5–14 min, 98–2% B1/B3; and 14–20 min, 2% B1 in positive mode or 14–17 min, 2% B3 in negative mode [31]. The mass spectrometer was operated in resolution mode (MSE) with electrospray ionization (ESI). The positive and negative ion mode spray voltages were set to 3.50 kV and 2.50 kV, respectively, and the sheath and auxiliary gases were 30 arb. and 10 arb., respectively [30]. Full scans were performed using a resolution of 70,000 in the scan range of *m/z* 81–1000 at a capillary temperature of 325 °C. Secondary lysis was performed using higher-energy C-trap dissociation (HCD) with a collision voltage of 30 eV, and dynamic exclusion of unnecessary MS/MS information was performed [15].

#### 2.4.3. Raw Data Analysis and Metabolite Identification

ProteoWizard software (v3.0.8789) was used for data acquisition [32]. The XCMS package of R (v3.3.2) was used for raw data analysis. Progenesis produced a feature matrix that contained accurate mass (*m/z*), retention time (RT), and chromatographic peak area. Parameters were set to obtain the data matrix. The data were normalized by total peak area normalization. The experimental and control groups subjected to mass spectrometry were scanned to collect data, and ions with the highest intensity were selected for continuous operation to obtain the base peak chromatogram (BPC). Sample quality control was performed using QC samples to remove poorly reproducible characteristic peaks (features) and optimize the data set for biomarker detection to achieve quality assurance (QA). Partial least squares discriminant analysis (PLS-DA) can better distinguish differences between groups and eliminate random errors unrelated to the purpose of the study [33], with the model cross-validation mainly referring to parameters R2X, R2Y, and Q2 [34]. R2X and R2Y represent the interpretability of the model in the X-variable and Y-variable data sets, respectively, and Q2 represents the predictability of the sample model; R2 and Q2 should be higher than 0.5.

For metabolite identification, the exact molecular weight of the metabolite was confirmed (molecular weight error, < 15 ppm), and fragmentation information was then obtained using the MS/MS model in METLIN (http://metlin.scripps.edu, accessed on 7 February 2022) and MoNA (https://mona.fiehnlab.ucdavis.edu//, accessed on 7 February 2022) to further match annotations and obtain accurate metabolite information. The obtained metabolic features were further screened to determine significance, and the metabolites were classified according to the criteria of *p*-value ≤ 0.05 and variable importance for the projection (VIP) ≥ 1 [35]. The identified metabolites obtained after identification of all measured molecular weights were functionally classified according to the metabolite classifications of KEGG and Metabolon, Inc. All metabolites identified after measuring molecular weights were classified, with the top 10 or 15 categories selected for pie charts.

#### 2.4.4. Validation Experiments for Target Metabolites with NIT Reduction Abilities

According to the significant relative content differences obtained by z-score and predictive analysis of the metabolite models and functions from the heat map, 11 metabolites were screened and selected as target metabolites for NIT degradation and formulated at a concentration of 10 mg/L for validation experiments [36,37]. The NIT content of the solution was measured after 24 h, and the NIT degradation histogram was plotted.

#### 2.4.5. Statistical Analysis

All experiments were performed in triplicate. The *L. fermentum* RC4 fermentation and MRS broths were divided into four portions of 25 mL and four portions of 37 mL, respectively. Multivariate statistical analysis, including principal component analysis (PCA), partial least squares discriminant analysis (PLS-DA), orthogonal partial least squares discriminant analysis (OPLS-DA) [29], and heat map analysis [38] were performed using R (v3.3.2). Self-adaptive (UV) conversion was used for data transformation. Features with *p*-value ≤ 0.05 and VIP ≥ 1 were considered significant.

The target metabolites were screened by combining the z-score score plot and differential metabolite heat map. The formula used to calculate the z-score was z = (x − µ)/σ. (x, µ, and σ correspond to specific scores, means, and standard deviations, respectively) [39].

Data were visualized by scaling to retain large differences, combining color gradients and using different color areas to represent different clustering group information, with columns representing samples, rows representing metabolites, and red and green colors representing experimental and control group samples, respectively. The magnitude of the relative content is shown in the graph by the shade of red and blue to plot a heat map of differential metabolites.

## 3. Results

### 3.1. Growth Curve and NIT Degradation Curve of L. fermentum RC4

**Figure 1:**
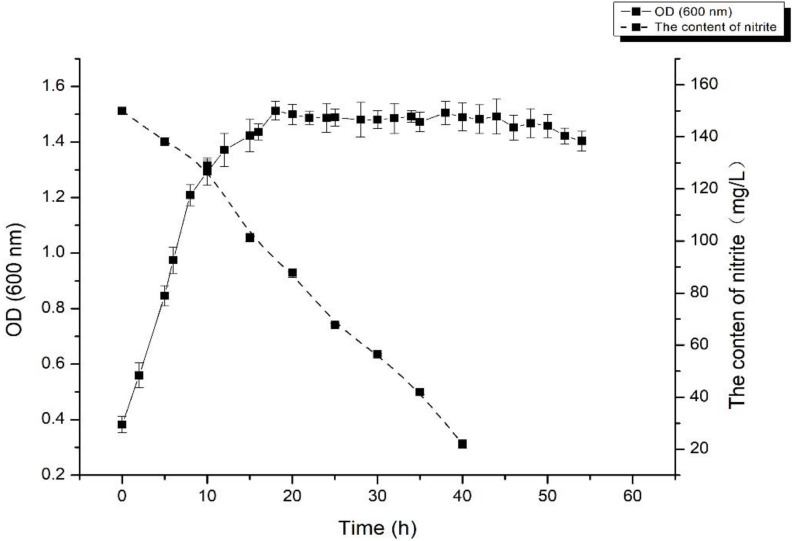
The growth curve and nitrite degradation curves for *L. fermentum* RC4.

As shown in Figure 1, the NIT degradation rate exerted by *L. fermentum* RC4 was relatively low at the end of the logarithmic growth period (15 h), with the NIT content decreasing from 150 mg/L to 101 mg/L at a degradation rate of 32%. After entering the stationary phase, *L. fermentum* RC4 produced a large number of metabolites, and the NIT content decreased significantly to 67 mg/L (25 h), with the NIT degradation rate reaching 55.3%. Finally, the NIT content decreased to 22 mg/L at 40 h, with a degradation rate of 85.3%, before entering the death phase. Therefore, degradation mainly occurred during the stationary phase.

### 3.2. Determining the Effects of Acids and Enzymes on NIT Degradation

**Figure 2:**
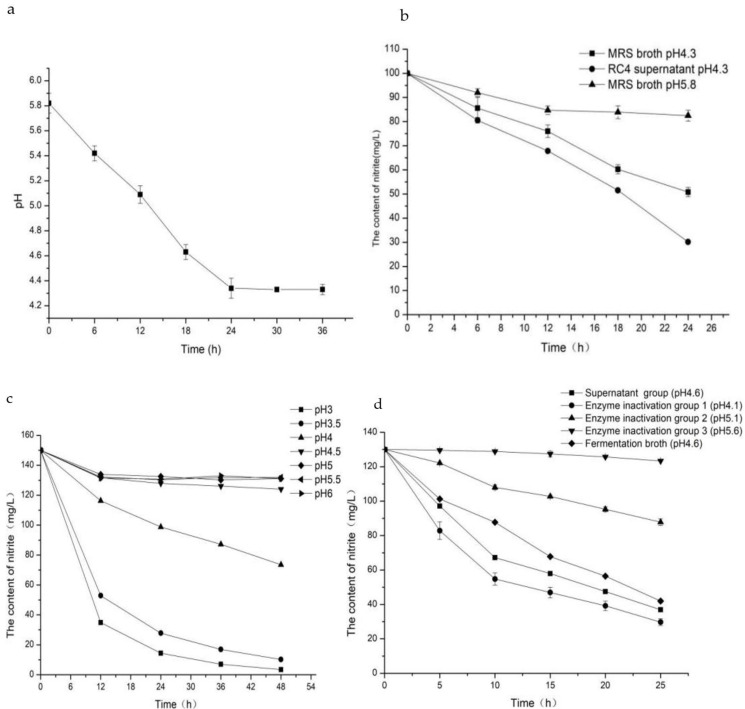
Acid production curves of *L. fermentum* RC4 (**a**) Nitrite degradation curves of *L. fermentum* RC4 metabolites and MRS culture broth; (**b**) Nitrite degradation curves in different pH; (**c**) Nitrite degradation curve of *L. fermentum* RC4 metabolites in synergy with acid (**d**).

The control group, MRS broth (unadjusted pH), had a weak degradation effect on NIT. The NIT degradation rates at 6-h intervals were 8%, 7.3%, 0.8%, and 1.4% (Figure 2b). Degradation mainly occurred in the first 12 h, after which the NIT content tended to be stable, and the NIT degradation rate was 17.5% after 24 h. After adjusting the MRS broth pH to 4.3, which was the same as the final pH of the RC4 fermentation broth (Figure 2a), the NIT content of MRS broth decreased from 100 mg/L to 50.8 mg/L, with a degradation rate of 49.2%, while the NIT degradation rate of *L. fermentum* RC4 metabolites at 24 h was 69.9%. The effect of different pH values on NIT degradation is shown in Figure 2c. At pH > 4.5, the NIT concentration in the solution showed little change, with a degradation rate of around 6%. At pH 4.5, the NIT content started to decrease, with the degradation rate reaching 17.3%. At pH < 4.5, the NIT degradation rate changed significantly, with degradation rates of 51%, 93.2%, and 97.7% at pH 4, 3.5, and 3, respectively. As shown in Figure 2d, the degradation pattern of RC4 metabolites showed that the NIT degradation rate of RC4 fermentation broth at pH 5.1 was 67.8%, while the NIT degradation rates of enzyme deactivation groups at pH 5.6, 5.1, 4.6, and 4.1 were 5.2%, 32.5%, 71.6%, and 76.9%, respectively. These results showed that NIT degradation by RC4 metabolites gradually increased with decreasing pH, confirming that the metabolites had a synergistic effect with acid in NIT degradation. Unexpectedly, the NIT degradation rate in the fermentation group without inactivation was 3.8% lower than that in the supernatant group, presumably due to the chemical reaction of NIT in the supernatant group caused by high-temperature inactivation.

### 3.3. LCMS Analysis for Screening and Identification of Target Metabolites for NIT Degradation

**Figure 3:**
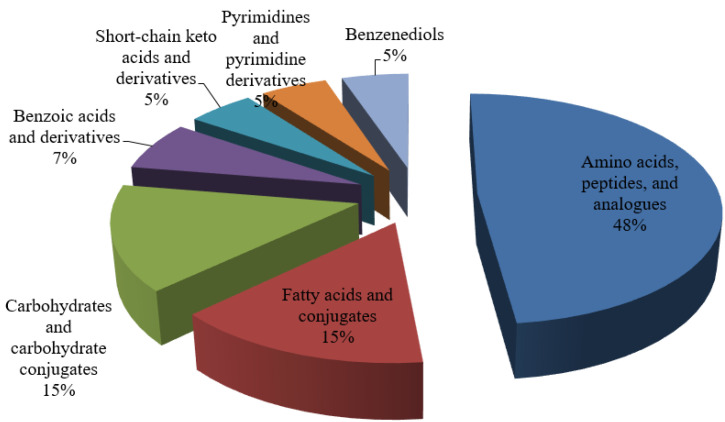
Metabolite full identification chemical structure subclass classification chart of *L. fermentum* RC4.

**Figure 4:**
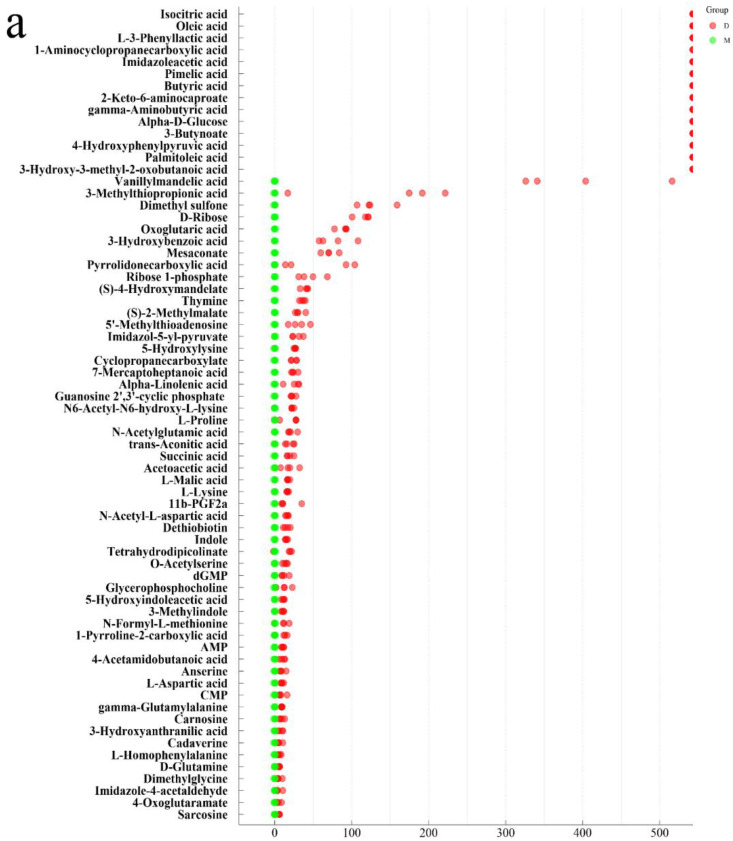

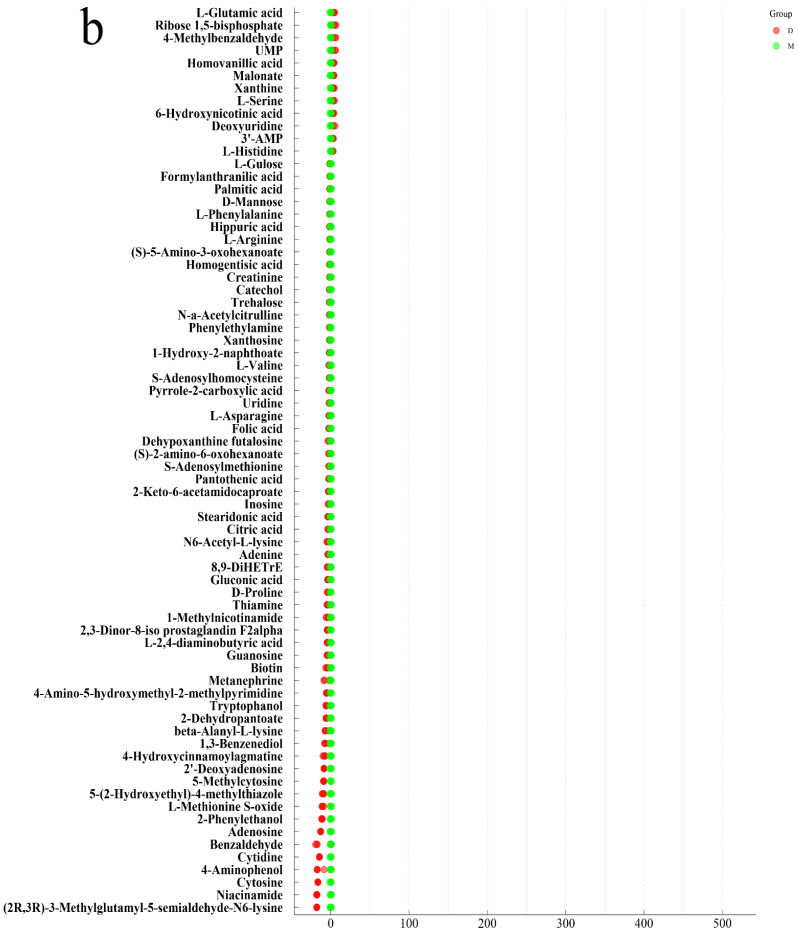
The z-score plot of the *L. fermentum* RC4 differential metabolites (**a**,**b**). Note—D: Experimental group; M: MRS broth group. The red and green dots represent the fermentation broth group and culture broth group, respectively, with four replicates in each group, and a greater distance between groups indicates the greater difference in relative content. As shown in Figure 4, the two groups were well aggregated within the group, and the difference between the groups was obvious.

**Figure 5:**
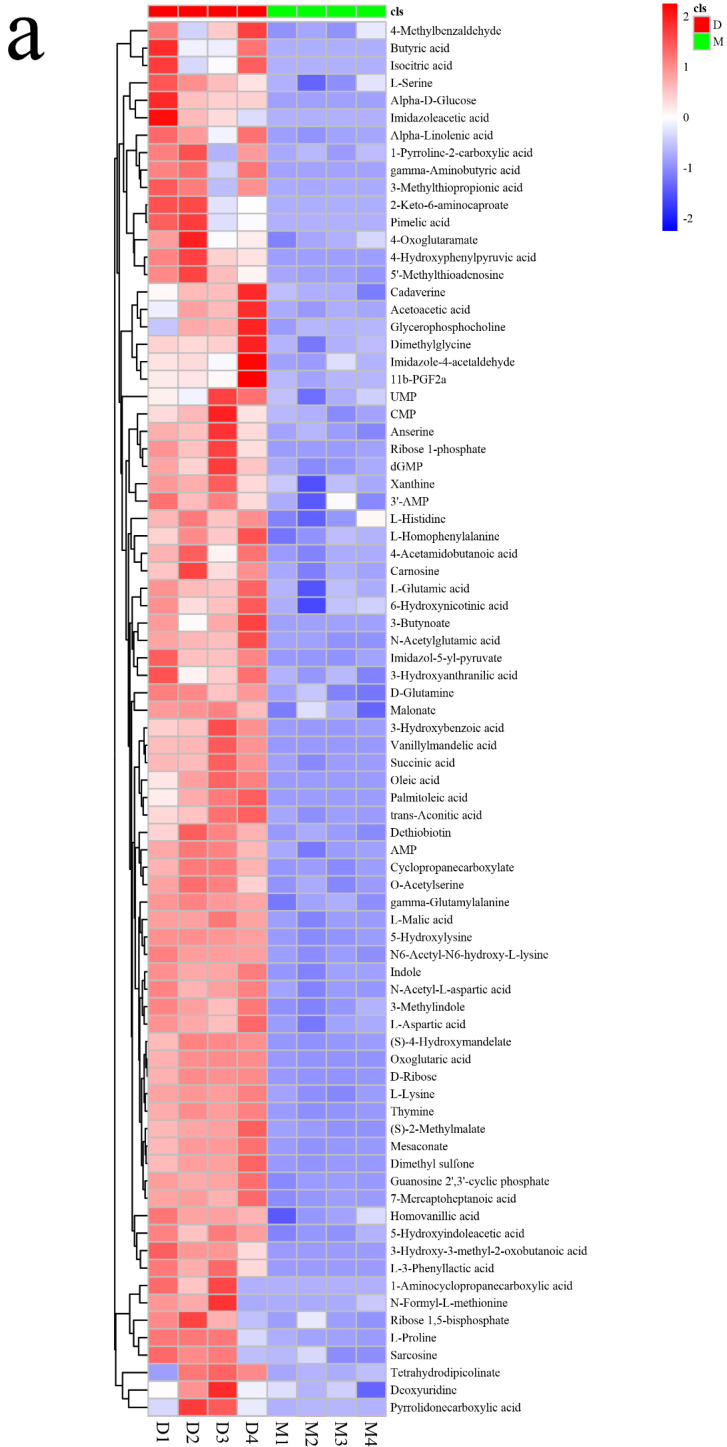

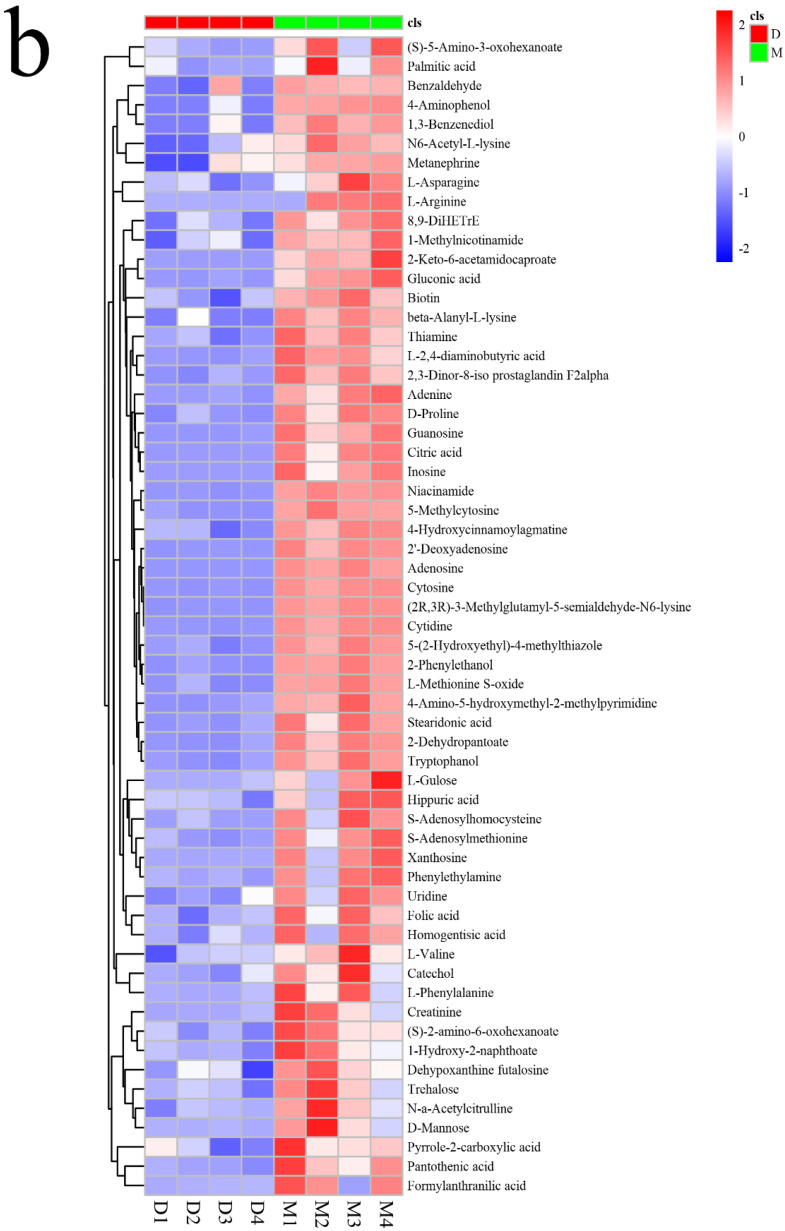
A heat map of *L. fermentum* RC4 differential metabolites (**a**,**b**). Note—D: Experimental group; M: MRS broth group. The magnitude of the relative content in the graph is shown by the different colors: red represents high sample content, blue represents low sample content, and the darker the color, the higher or lower the relative content. The columns represent sample groups and the rows represent metabolites. Both fermentation broth and culture broth groups were divided into four replicates: D1, D2, D3, and D4, and M1, M2, M3, and M4, respectively. Two metabolite groups were clustered within the group, and the metabolic patterns or metabolic pathways were distinct between the groups.

Non-targeted LCMS analysis was performed on the *L. fermentum* RC4 fermentation broth and MRS broth (without fermentation) culture and fermentation broth, with 43,837 metabolic signatures observed. To reduce errors and ensure the identification of differential metabolites, further QA and QC treatments were performed; PLS-DA and substitution tests were used to ensure the quality of the data model. The QA-treated samples were better aggregated, and the proportion of QC samples with RSD < 30% was around 70%, which ensured the data quality [34]. The samples were clustered within groups and discrete between groups, with R2X and R2Y values of 0.528 and 0.999, respectively, and a Q value of 0.935. Furthermore, a permutations plot for PLS-DA was used to assess the overfitting problem of the model. The rightmost Q2 values of the experimental model were all greater than the left Q2 values, indicating that the model had a good predictive ability [40]. A total of 305 metabolites were detected in the experimental and control groups (Figure 3). Among them, 140 differential metabolites were detected and classified by chemical structure. These included 67 amino acids, peptides, and analogs; 21 fatty acids and adducts; 21 carbohydrates and carbohydrate adducts; 10 benzoic acids and their derivatives; 7 short-chain keto acids and their derivatives; 7 pyrimidines and pyrimidine derivatives; and 7 benzenediols. Among them, 134 metabolites had significantly different contents. Based on the relative contents of metabolites and the mean and standard deviation of the reference data set (control), metabolite z-score plots (Figure 4) and a heat map (Figure 5) were constructed to determine the high and low relative metabolite contents at the same level. Eleven types of target metabolites for NIT degradation were screened (Table 1).

### 3.4. Validation Experiments of Target Metabolites for NIT Reduction

**Figure 6:**
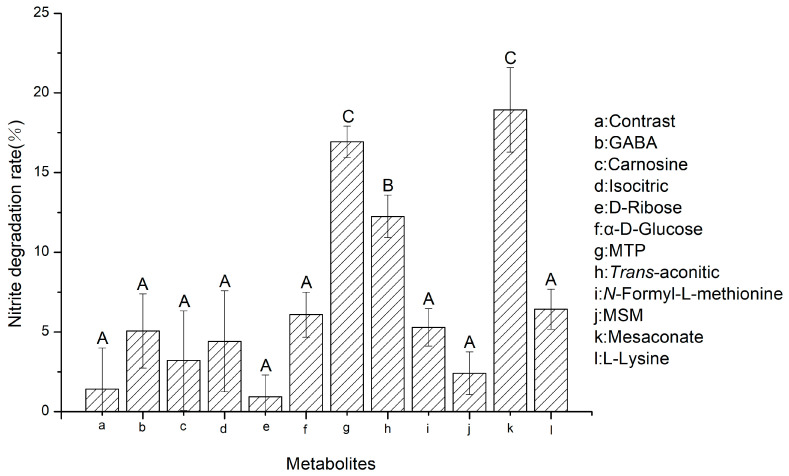
A histogram of nitrite degradation by target metabolites in 24 h. Note: A-C represent that there are significant differences between metabolites with different letters, *p* ≤ 0.05.

As shown in Figure 6, validation experiments were conducted on the metabolites for NIT degradation screened in the previous experiments. The results showed that the natural NIT degradation rate in the control group after 24 h was 1.41%, while the corresponding NIT degradation rates of mesaconate, MTP, *trans*-aconitic acid, L-lysine, N-formyl-L-methionine, MSM, D-glucose, GABA, isocitric acid, carnosine and D-ribose were 18.93%, 16.93%, 12.25%, 6.42%, 5.28%, 2.41%, 6.08%, 5.07%, 4.41%, 3.21%, and 0.93%, respectively. This suggested that mesoconate, 3-methylthiopropionic acid and *trans*-aconitic acid had better NIT degradation effects.

## 4. Discussion

Acid degradation of nitrite must be lower than a certain pH (pH 4.5 was the critical acid level for NIT degradation), and acid degradation gradually becomes the main factor of nitrite degradation at pH < 4.0 [9]. However, in this study, the pH of RC4 fermentation broth was higher than 4.0 throughout the process exhibiting efficient degradation, showing that the acid degradation is not the main factor. The degradation mechanism of nitrite by non-acid and non-enzyme substances of lactic acid bacteria was explored in this work. Meanwhile, MRS broth contains many types of substance, such as peptone and beef paste, that will become more complex after autoclaving and making NIT degradation possible. This hypothesis was confirmed, and the MRS broth was found to have a synergistic effect with acid. Compared with MRS broth, the RC4 metabolites were proven to degrade NIT faster and to a greater extent. At an initial NIT concentration of 130 mg/L, the NIT degradation rate of the RC4 metabolites adjusted to pH 4.1 reached 77.4%. Furthermore, the degradation effect was much better than that of acidic buffer solution at pH 4, indicating that RC4 metabolites played an important role in the NIT degradation process. Further analysis of the RC4 metabolites showed that these substances had a synergistic effect with pH, similar to previous studies on the degradation of nitrite by protein and acidic polysaccharide [19,22].

A non-targeted metabolomics approach was used to screen and identify the RC4 me-tabolites, with 11 metabolites exhibiting potential NIT degradation abilities obtained according to the z-score plot and the heat map. Isocitric acid and citric acid are organic compound substrates involved in the tricarboxylic acid cycle [41]. Among these, isocitric acid has antiox-idant effects; it can oxidize and decarboxylate to α-ketoglutarate to be used as a substrate for NADH and NADPH under the action of isocitrate dehydrogenase [42], and NADH is an electron donor for NirBD [13]. Although isocitric acid alone has a limited NIT degradation ability, it can promote the enzymatic degradation of NIT by RC4 in an NADH-providing manner [43]. A similarity was observed among mesaconate, MSM, lysine, and D-ribose. Mesaconate is an unsaturated dicarboxylic acid with a methyl-branched chain commonly found in Clostridium tetanomorphum [44] and Burkholderia xenovorans [36]. It is an intermediate in the glutamate degradation pathway, and it can be further converted into pyruvate and acetyl-CoA by undergoing decarboxylation reactions [45,46]. In this study, mesaconate showed a significant difference between the experimental group and the control group, and the results of the validation experiments showed that it has a strong NIT degradation ability, which is presumed to be related to its chemical structure. This is subject to further quantitative analysis. Lysine can provide energy for biological metabolism and promote glucose synthesis [47]. As among the most widely distributed and important monosaccharides in nature, glucose is used as a reducing and concentrating agent for the chemical properties of aldose [48]. Ma et al. [49] found that glucose as a substrate can promote denitrification to remove nitrate nitrogen in denitrifying bacteria and anammox bacteria. The results showed that the relative content of D-glucose in metabolites was significantly upregulated, which could promote the growth and reproduction of RC4 and improve its NIT degradation ability. D-ribose is a pentose monosaccharide containing five carbon atoms that have a metabolic model and functional prediction similar to those of mesoconate and L-lysine, among others (Figure 4). Ribose has been reported to have reducing properties owing to the presence of free aldehyde groups in its molecular structure [50]. However, validation experiments showed that ribose had little degradation effect, which might be due to the low addition level. Furthermore, GABA showed a clear decreasing trend compared with the control group; research has suggested that GABA is a non-protein aminotetracarbonate widely distributed in a free state in prokaryotes and eukaryotes [51]. GABA is an antioxidant that promotes enzymatic activity and the synthesis of other antioxidants, such as citrate, as a metabolite indirectly involved in NIT degradation by RC4. Furthermore, an increased content of N-formyl-L-methionine was observed in the experimental group. This compound contains three functional groups, namely, carboxyl, amino, and methylthio moieties, and is involved as an important intermediate metabolite in the synthesis of methionine [52]. Methionine is an essential amino acid for the growth of both animals and bacteria, and it involved in both biosynthetic and regulatory processes in the bacterial cell. Methionine also participates in the methionine cycle, which carries out synthetic and regulatory methylation reactions [53]. The increase in N-formyl-L-methionine shows that the metabolic activity of lactic acid bacteria is enhanced, which is helpful to promote the degradation of nitrite. MSM, which is reported to have antioxidant effects, such as scavenging oxygen radicals, showed a slight increase in the experimental group [54]. Furthermore, carnosine is a dipeptide synthesized from β-alanine and L-histidine, and is a non-enzymatic free-radical scavenger and a natural antioxidant [55]. It exhibits pH-buffering activity [56], heavy metal chelating activity [57], and anti-glycating activity [58]. Carnosine reduces lipid peroxidation, but also inhibits the oxidative modification of protein exposed to hydroxyl radicals [59] and is involved in the nitrosative stress and heat shock pathways [60]. In addition to mesaconate, *trans*-aconitic acid and MTP were determined to be effective metabolites for NIT degradation (Figure 3). *Trans*-aconitic acid is an unsaturated tricarboxylic acid containing a double bond that is readily decarboxylated to form a clathrate. Owing to the negative charges conferred by its carboxylic groups, *trans*-aconitic acid can also act as a divalent cation chelator. MTP also contains carboxyl groups. A variety of metabolites promote the degradation of nitrite, and the *trans*-aconitic acid and MTP were speculated to be the important metabolites of RC4 for NIT degradation, with the degradation mechanisms perhaps being related to decarboxylation reactions. Herein, a preliminary profile was conducted for NIT degradation by non-acid and non-enzyme metabolite substances of lactic acid bacteria. The specific components of LAB metabolites with NIT degradation ability will be used to improve the safety and flavor of pickled and cured foods.

## 5. Conclusions

*L. fermentum* RC4 degraded NIT by producing nitrite reductase and decreasing the pH of the system during fermentation. Furthermore, substances produced by RC4 metabolism exhibited strong NIT degradation abilities and were more effective when synergized with acid. According to untargeted metabolomics analysis of the RC4 metabolites based on LCMS, 134 significantly different metabolites were identified. By analyzing their relative contents and predicting their metabolic functions, 11 target metabolites for NIT degradation were identified. Verification showed that GABA, isocitric acid, D-glucose, MTP, N-formyl-L-methionine, MSM, D-ribose, mesaconate, *trans*-aconitic acid, L-lysine, and carnosine showed significant NIT degradation effects compared with the control group (MRS broth), with NIT degradation rates of 5.07%, 4.41%, 6.08%, 16.93%, 5.28%, 2.41%, 0.93%, 18.93%, 12.25%, 6.42%, and 3.21%, respectively. Among these, mesaconate, MTP, and *trans*-aconitic acid exhibited the strongest NIT degradation abilities, with degradation rates of 18.93%, 16.93%, and 12.25%, respectively. Furthermore, RC4 metabolites, including isocitric acid, lysine, glucose, D-ribose, GABA, and carnosine were able to indirectly degrade NIT by supplying energy, promoting the synthesis of antioxidant substances, and promoting enzymatic activity, resulting in the ability of RC4 to efficiently degrade NIT. In this study, a new pathway for NIT degradation by LAB metabolites other than enzymatic and acid degradation was analyzed using metabolomics for the first time, which is beneficial for addressing the hazards posed by excessive NIT in food.

## Figures and Tables

**Table 1:** The key nitrite-degrading compounds screened from the *L. fermentum* RC4 fermentation broth.

Metabolites	VIP	Mean ± Sd_D * 10^7^	Mean ± Sd_M * 10^5^	Fold Change	*p*	Trend
GABA	1.372	115.22 ± 58.87	0. 09 ± 0	128,740.00	0.008	↑
Isocitric acid	1.198	1.03± 0.78	0. 004 ± 0	27,749.00	0.038	↑
D-Glucose	1.430	3.89 ± 1.69	0.09 ± 0	4343.70	0.004	↑
MTP	1.309	90.55 ± 54.25	100.72 ± 59.23	89.90	0.016	↑
*N*-Formyl-L-methionine	1.176	56.56± 41.75	286.89 ± 519.96	19.72	0.043	↑
MSM	1.582	253.59 ± 40.37	1905.95 ± 183.30	13.31	0.000	↑
D-Ribose	1.608	436.95 ± 34.55	3660.9 ± 347.31	11.94	0.000	↑
Mesaconate	1.594	147.49 ± 18.76	1254.64 ± 190.24	11.76	0.000	↑
*Trans*-aconitic acid	1.529	38.15 ± 10.14	386.58 ± 174.62	9.87	0.001	↑
L-Lysine	1.607	1943.68 ± 93.41	60,314.90 ± 8086.20	3.22	0.000	↑
Carnosine	1.489	228.88 ± 47.77	9040.00 ± 1544.90	2.53	0.001	↑

Note: *—VIP, variable importance for the projection; GABA, Gamma-aminobutyric Acid; MTP, 3-methylthiopropionic acid; MSM, dimethyl sulfone; Mean ± Sd: Average value and standard deviation of the peak area in substance in mass spectrometry. D and M, respectively, represent *L. fermentum* RC4 fermentation supernatant and MRS broth; Fold change (FC) represents the degree of variation between experimental control groups and is a key factor in screening for differential metabolites, with FC ≥ 1.5 or FC ≤ 0.667 indicating large differences in content between the two groups; *p*, statistical *p*-value; “↑” indicates that the content of the test group increases.

## Data Availability

Data is contained within the article.
